# Facilitating the Development of Deep Learning Models with Visual Analytics for Electronic Health Records

**DOI:** 10.3390/ijerph17228303

**Published:** 2020-11-10

**Authors:** Cinyoung Hur, JeongA Wi, YoungBin Kim

**Affiliations:** 1 Linewalks, 8F, 5, Teheran-ro 14-gil, Gangnam-gu, Seoul 06235, Korea; cinyoung.hur@linewalks.com; 2 Graduate School of Advanced Imaging Science, Multimedia & Film, Chung-Ang University 84, Heukseok ro, Dongjak-gu, Seoul 06974, Korea; placeja@cau.ac.kr

**Keywords:** electronic health records, visual analytics, deep learning models

## Abstract

Electronic health record (EHR) data are widely used to perform early diagnoses and create treatment plans, which are key areas of research. We aimed to increase the efficiency of iteratively applying data-intensive technology and verifying the results for complex and big EHR data. We used a system entailing sequence mining, interpretable deep learning models, and visualization on data extracted from the MIMIC-IIIdatabase for a group of patients diagnosed with heart disease. The results of sequence mining corresponded to specific pathways of interest to medical staff and were used to select patient groups that underwent these pathways. An interactive Sankey diagram representing these pathways and a heat map visually representing the weight of each variable were developed for temporal and quantitative illustration. We applied the proposed system to predict unplanned cardiac surgery using clinical pathways determined by sequence pattern mining to select cardiac surgery from complex EHRs to label subject groups and deep learning models. The proposed system aids in the selection of pathway-based patient groups, simplification of labeling, and exploratory the interpretation of the modeling results. The proposed system can help medical staff explore various pathways that patients have undergone and further facilitate the testing of various clinical hypotheses using big data in the medical domain.

## 1. Introduction

Improving the quality of medical treatment and hospital administration by gaining insight from a massive amount of clinical information is a critical issue in healthcare. In particular, massive amounts of data have been accumulated in hospitals since electronic health records (EHRs) became ubiquitous. Many hospitals gather and utilize voluminous data, thereby facilitating big data mining and deep learning techniques to tackle diverse medical issues [1,2]. Especially, many research methods of cardiovascular disease have attempted to improve patients’ outcomes, such as early diagnosis of heart failure, by aggregating millions of patients’ EHRs from various resources [3].

Although deep learning-based models have exhibited excellent performance in healthcare, it is challenging to develop novel models for various reasons, such as the necessity of medical and engineering domain knowledge, complex working definitions of clinical outcomes, and the requirement of iterative experiments for achieving higher performance. Many studies have attempted to facilitate the development process using visual assistance; however, this is still a challenging task. In particular, few studies have attempted to assist in cohort definition, EHR labeling, and debugging deep learning models [2].

To address these challenges, EHR mining has been applied to reveal patient stratification and unknown disease correlations [4]. Many studies have been conducted to visualize the results of medical sequence patterns or various treatment behaviors extracted from large-scale EHR data [5,6,7,8]. Furthermore, previous papers have reported techniques for visualizing frequent events [9] and for sorting and visualizing events by stage to understand disease progression for care plans [10,11,12,13]. Another technique summarizes data into multiple sequential patterns and then presents them visually [14]. Both data mining and visualization have been used to assist in patient extraction and statistical analysis [13,15]. However, previous studies mainly focused on the exploration of EHR data, and more studies are required for incorporating EHR mining results with the development of deep learning models.

In addition, various types of data generated by models, such as deep learning model data (e.g., hidden layer weights), need to be explored in detail for model debugging and for interpreting the predicted outcome. A visual approach was previously used for debugging machine learning models [16,17,18]. However, a new approach is still needed because the medical field requires sufficient exploration of data during the development of reliable predictive models.

In the present study, we combine data mining and visualization to increase the efficiency of using EHR data. We propose an interactive visualization system to help engineers and medical staff seek pathways of interest and access the prediction results of deep neural networks for EHR data. By selecting clinical pathways in the visualization system, patient groups are extracted and labeled without directly analyzing complex EHRs. In addition, we propose visual assistance, which can present variables with high attention weights. These weights were determined by training a deep neural network model and attention mechanism.

## 2. Materials and Methods

### 2.1. Data Description and Cohort Construction

We used the MIMIC-IIIdatabase released by the MIT Lab for Computational Physiology. This database contains 61,532 medical records involving intensive care unit (ICU) treatment [19]. All data were extracted from the database by using the standard Structured Query Language (SQL).

To experiment model development for cardiovascular disease, we chose patients who were diagnosed with cardiovascular diseases. From data on all the inpatients, we extracted data for a total of 11,811 adults. The cardiovascular diseases are given the following code names based on the International Classification of Diseases, Ninth Revision (ICD-9): (1) congestive heart failure, 428.0–428.4, 428.9, 398.91; (2) ischemic heart disease, 410–414; (3) myocardial infarction, 410; and (4) other cardiomyopathies, 425.

We created two target variables for individual inpatients. The first variable is heart failure, which corresponds to congestive heart failure and is defined in ICD-9, and the second is an unplanned cardiac surgical treatment that is referred to as coronary artery bypass grafting (CABG) or percutaneous coronary intervention (PCI).

From the MIMIC-III database, we collected data on administration, diagnosis, comorbidity, ICU, ICU service, laboratory tests, prescription, and procedure from the cardiovascular cohort. This cohort and detailed data were chosen to highlight correlations among lab test results, vital signs, medication, and crucial medical events. The event definition of the dataset is presented in the Supplementary Materials.

The records that do not correspond to hospitalization were removed such that we included only those that were created during hospitalization. When the time of hospitalization was subsequent to discharge or death, the record was regarded as an error and eliminated. To aid the reproduction of this study, data preprocessing codes and analysis codes will be made available on GitHub (https://github.com/linewalks/EXI-Paper).

### 2.2. Architecture

**Figure 1:**
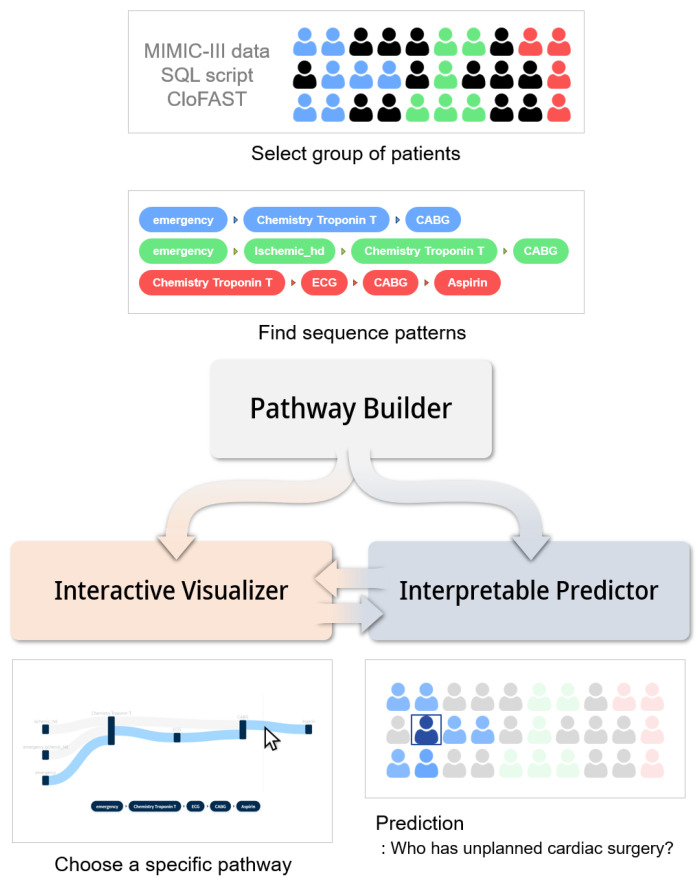
System architecture. CABG, coronary artery bypass grafting.

Figure 1 shows the system architecture designed in this study. It consists of three key elements. The pathway builder converts MIMIC-III data into a structure for which a sequence pattern is easy to find with the preprocessing SQL script. The sequence pattern mined using the CloFASTalgorithm, a closed sequential pattern mining technique, is used to select a group of patients corresponding to clinical events of a specific sequence [20]. The interpretable predictor predicts the probability of occurrence of heart failure or heart surgery when a patient is hospitalized. In addition to the probability, it provides the weight of each variable computed using the attention mechanism. The visualizer is developed as a web-based application using ReactJS, a JavaScript library for building user interfaces. It serves as a user interface and facilitates fine-grained constraints of cohort selection by providing an interactive interface to choose a specific pathway in a merged pattern graph. Moreover, it offers an interface that makes it easy to search for the weight of a variable found in the interpretable predictor.

### 2.3. Sequence Mining for Clinical Pathways

We applied domain knowledge to data preprocessing, instead of using raw data for learning. In preprocessing, three crucial aspects were considered in medical events.

- Context: Important events differ depending on the context. Each disease has a different important event, which implies that every event must not be treated equally and must be included or excluded according to the purpose. For example, for the use case presented herein, it is possible to include echocardiography, which is strongly related to heart diseases.
- Granularity: Medical codes have an intrinsic classification scheme, which implies that the whole hierarchy must be adjusted according to the purpose. For example, it is possible to analyze even the lowest level of disease codes in detail and divide it into efficacy groups by using medical codes.
- Irregularity: The cycle of event occurrence has irregularity, which increases the computational cost and negatively affects learning performance. Therefore, it is necessary to assemble data per hospitalization unit or day.

**Figure 2:**
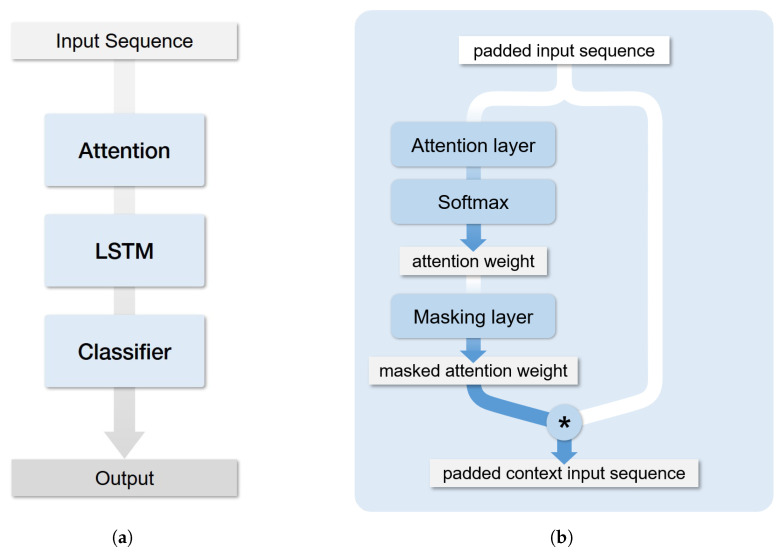

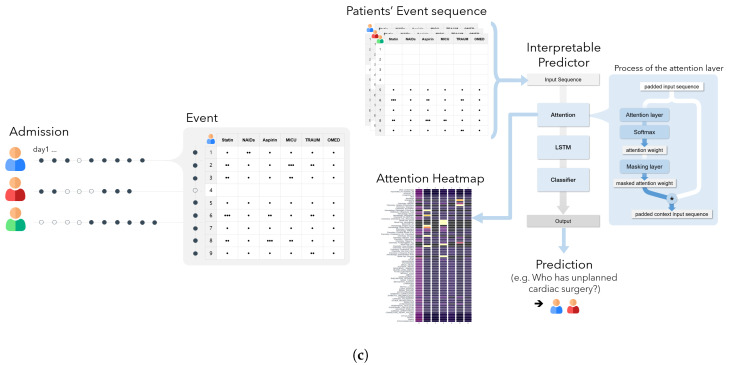
Architecture of the long short-term memory (LSTM) attention model. (**a**) The overall structure of the LSTM attention model and (**b**) the detailed process of the attention layer. Further, (**c**) shows the entire modeling process, including the data input and output.

**Figure 3:**
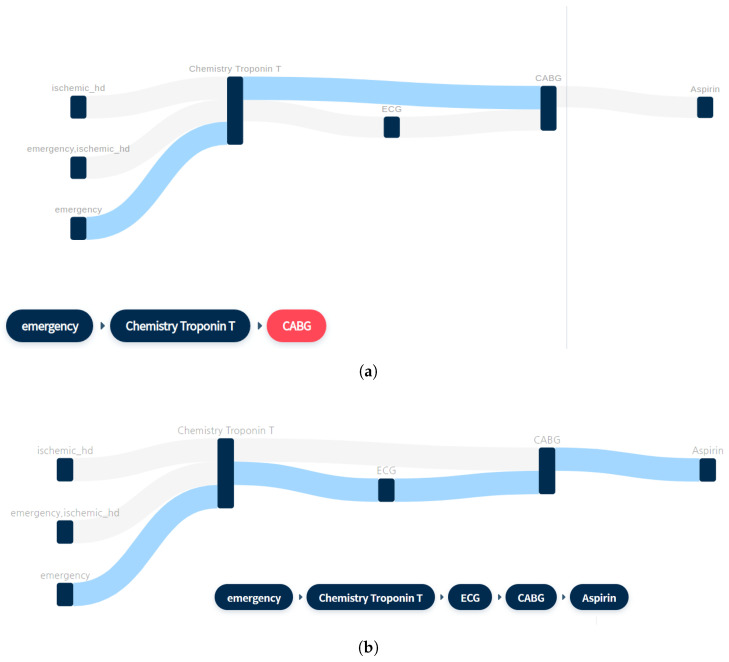

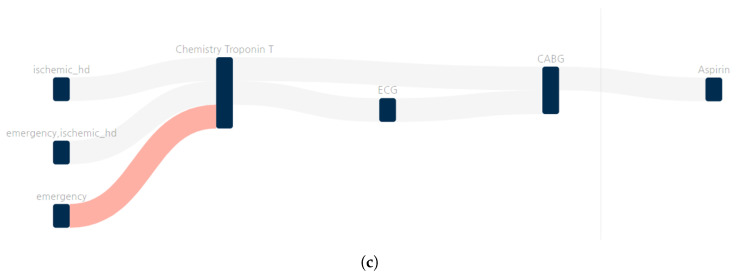
Example of a clinical pathway. (**a**) Example of a flowchart for sequenced events. (**b**) When an event is selected in the normal order, the link is highlighted in blue, and the selected card is displayed below. (**c**) When the event is selected in reverse order, it is highlighted in red, and the selected card is not displayed.

As exemplified in Table 1, this study organized sequential data by extracting patient IDs, event timestamps, and event values from EHR data in order to search for patients’ treatment pathways using sequential data. The CloFAST algorithm was used to find the pattern that best represents the pathways through which many patients have gone. The mining results are used and visualized as a clinical pathway, as shown in Figure 2c and Figure 3.

For utilizing clinical information extractable from EHR data, the proposed system provides a customized level of detail according to the types of medical events, time intervals, and order. These parameters enable users to find a group of patients with a particular sequence of patterns.

### 2.4. LSTM Attention Model

An RNN is useful for processing sequential data. Vanilla RNN is difficult to train with long sentences because of problems such as gradient vanishing or gradient exploding. LSTM undoubtedly has an advantage over RNNs for learning long-term dependencies. LSTM allows networks to learn long-term dependencies by using various types of gates to control information flow.

It is difficult to explain the intermediate steps and results of LSTM, as with other deep learning models. In this study, to overcome such difficulties in interpretability, we adopted the attention mechanism, which makes a model focus on specific variables in given sequences.

To ensure the interpretability of the levels of the input variables, the weights, $W_{k}$, were learned and used in computing the attention vectors, $a_{k}$, for the features of each time step, $x_{k}\cdot a_{k}=softmax\left(W_{k}\cdot x_{k}\right)$, where each $x_{k}$ represents a feature of the time steps. Before data are used as input to the LSTM, input features in the form of time series are given with weights by the learned attention.

In this study, LSTM was used to predict two use cases: the diagnosis of congestive heart failure and the probability of unplanned cardiac surgery. The first use case was implemented using PyTorch, while the second used Keras with the TensorFlow backend. Data used as input to the model were a three-dimensional time series consisting of the patients’ hospitalizations (*n* = 22,495), time steps (*n* <= 15), and features (*n* <= 251). An example of the data employed is provided in Figure 2c. These time series were used as input to the attention layer described above. Because the number of days of hospitalization differed among patients, the length of the entire sequence was truncated. For example, if the length of the sequence is specified as 15, the sequence is deleted when the number of hospitalization days is greater than 16. Likewise, padding is performed when the number of hospitalization days is less than 15. Sequences padded with zeros are fed to the mask layer, which ignores the attention weights.

Finally, the output of the masking layers was used as the input to the LSTM layer. The LSTM layers consisted of 128 units. The output layer of the models was a dense layer with sigmoid activation, which output the probability of a given event. Activation maps consisting of softmax activation, ak, for each input variable were extracted from the attention layer to interpret the model output. The structure of the model is summarized and described in Figure 2.

Before the hyperparameter optimization of the models with the structure described above, datasets were divided into training, validation, and test datasets in proportions of 0.7, 0.15, and 0.15, respectively. The number of units in the LSTM layer was set to be equal to or less than the number of features. All hyperparameters were selected before model training and adjusted after training according to the accuracy of the model for validation data and the area under the receiver operating characteristics (AUROC). The model was trained by the adaptive moment estimation (Adam) optimizer with a learning rate of 0.001, beta1 of 0.9, beta2 of 0.999, and epsilon of 1 $\times \;10^{-8}$. The batch sizes were set to 64 and 256 for individual use cases. The model was trained for 10 and 20 epochs, respectively. The LSTM attention model was cross-validated using datasets split into a ratio of 7:3; moreover, it achieved AUROC and F1 values of 0.9704 and 0.8156, respectively, for the test dataset. Under the same conditions, the RNN attention model achieved AUROC and F1 values of 0.91 and 0.59, respectively, for the test dataset.

Two types of visualizations were used in this study: flowcharts for sequenced events such as clinical pathways and heat maps for visualizing the magnitude of the weight for each time step and variable. All charts were made interactive to help with operation and search. We interpreted the patient group selection as a series of consecutive event selections. Many clinical studies select subjects based on a series of events; for example, selecting a group of patients who have been diagnosed with a specific disease and have taken a specific drug. Each event has an order and is merged into one event or branched into multiple events over time. Furthermore, the number of times two events occur in succession varies. The flowchart was chosen as a visual encoding that expresses these characteristics well. As described in Figure 3, when a sequence of events is selected, a link that shows the event’s order is highlighted. This interaction was used to define patient groups corresponding to the pathway selected by the user, which provides a much more intuitive and quicker means than querying the corresponding number of patients in SQL.

**Figure 4:**
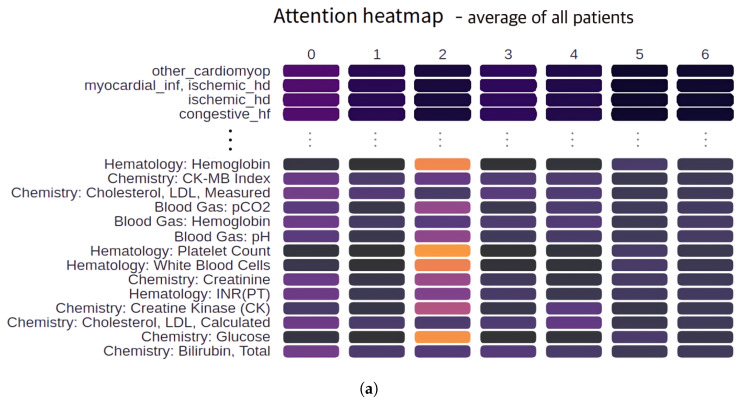

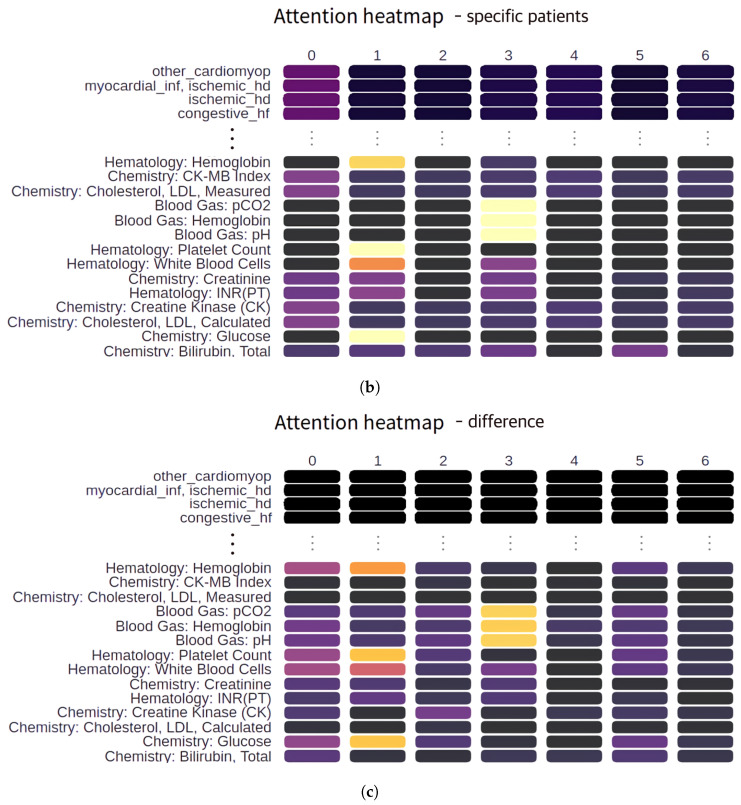
Attention heat maps. (**a**) Average attention for all patients in the case of cardiac surgery prediction in hospitals, (**b**) variableweights corresponding to specific patients, and (**c**) difference between the average weight for the patient group and variable weights corresponding to specific patients.

The heat map was used as a visual analysis tool that supports variable-level weight search with the debugging of trained models, as shown in Figure 4. Nodes in the heat map correspond to variables of a specific time step, and the node color represents the weight. A change in color in the horizontal direction indicates a change in the weight of the variable over time. The color difference in the vertical direction makes it possible to compare the weights of variables in the same time interval. A controller that filters variables with weights above a threshold and interactions such as mouseover and mouseleave was used to assist the search of the daily weights of variables of the multi-dimensional LSTM.

## 3. Results

As an example, we considered the case of training a model to predict the probability of unplanned cardiac surgery. Although conditions that require cardiac surgery may vary, we focused on patients who followed a series of events as a high-risk group: patients undergoing CABG surgery after an emergency hospital admission and elevated troponin levels. We implemented this example as a web-based application (https://exi-paper.herokuapp.com/).

Figure 1 shows a clinical pathway extracted from the pathway builder (see Materials and Methods for details on the architecture used). The pathway includes the events before and after the event of interest. By using the interactive function in the system, users can easily search for patterns of interest and gain insight.

Sequence mining used data from a total of 22,495 hospitalization cases for 11,811 patients (excluding scheduled outpatients). The mining was performed for emergency and urgent cases among hospitalization events and the troponin test among diagnosis events, including the prescription group, procedure, comorbidity, and diagnosis categories. The original timestamps of events were used. The minimum support rate in mining was set to 25, 10, 5, and 3%, and the pattern resulting from the case of 3% was explored. Although sequence mining is frequently used with high support, we chose this low support rate because most label ratios tend to be very imbalanced in practice. As summarized in Table 2, a lower support rate is associated with a longer total running time and a greater number of found patterns. Accordingly, the minimum support value was empirically determined. Most serious medical outcomes occur rarely. Therefore, it was necessary to perform decisions empirically by setting a sufficiently small support to include the desired outcomes in the discovered pattern.

Table 3 lists the mined patterns that are strongly related to unplanned cardiac surgeries. A total of twelve patterns were found, among which the pattern of primary interest was pathway no. 4.

Figure 4a shows the averaged attention heat map for 22,495 patients. The heat map helps in identifying variables that are common to patients. Overall, the variables on the first day of hospitalization are the most important. The user can check the weight for each variable through hovering and choose to display only the variables above a certain weight through the filtering option.

Figure 4b visualizes the variable weights for patients who underwent surgery on the second day of hospitalization. From Day 1, the weights of the diagnostic tests for fibrinogen, hemoglobin, platelet count, glucose, and urea nitrogen increased. Figure 4c visualizes the average variable weights for a group of patients versus the variable weights for specific patients.

**Figure 5:**
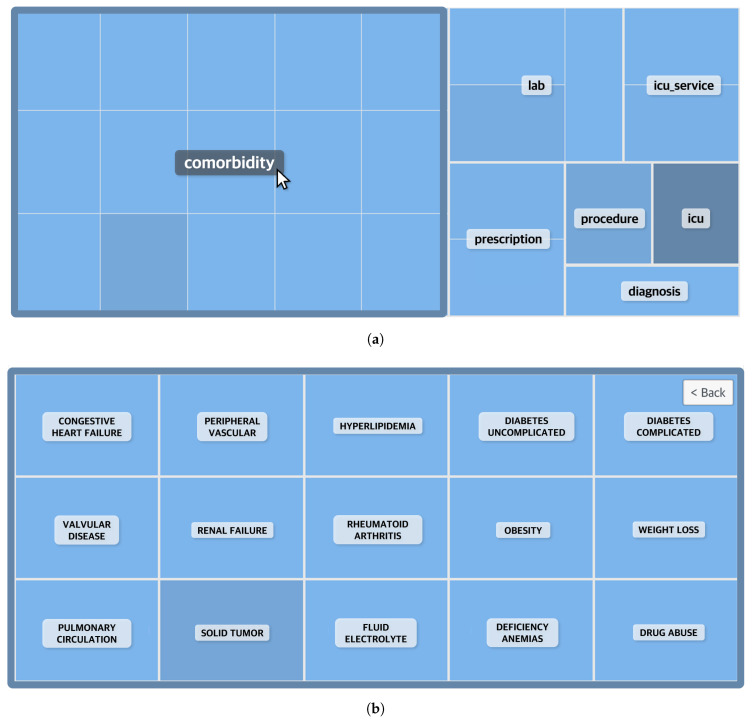
Visualization of the top 25 attention values of the in-hospital congestive heart failure diagnostic prediction model with a TreeMap. (**a**) Events are grouped by category. Selecting a category shows the name of the detailed events. (**b**) A detailed event screen that appears when the category “comorbidity” is selected.

Figure 5 depicts the visualizations of the attention of the in-hospital congestive heart failure diagnostic prediction model in a heat map. Of all the patients, fifteen percent were sampled and schematized. The top four variables noted in the model are first, medical intensive care unit (MICU); second, echocardiography; third, diagnosis of solid tumor complications; and fourth, cholesterol level in the diagnostic test. Selection by priority and provision of the variables to be reviewed by the medical staff, as shown in this example, can contribute to reducing the cognitive burden on the medical staff.

We conducted a case study for the qualitative evaluation of the proposed method. Expert users who participated in this case study took part in follow-up interviews to provide qualitative feedback. User1 is a cardiologist, whereas User2 and User3 are internists. Their comments are summarized below. User1 said that it would be excellent if the development pipeline proposed in this study can be applied to the hospital information system in real time. In addition, he noted that the proposed pipeline would be helpful to clinicians developing a clinical predictive model with regard to defining outcomes that entail complex clinical criteria. User2 said that the Sankey diagram could assist in selecting sub-populations by selecting events.

Moreover, User1 mentioned that the analysis of the deep learning model, which is a black box, is necessary; nevertheless, the performance of the model is much more important. When the performance is high and the model is well calibrated, model interpretation is not always required. Further, he mentioned that “variable attention does not support decision making. For example, an ECG test is essential for patients with heart disease, but it was not possible to determine whether it was recommended or not. However, during model development, clinicians have a low understanding of the process by which the model is developed or data are queried using SQL; thus, visualization would be of significant help.” User1 believes that the generated heat map is informative and creative as it aids in time series interpretation of EHR data. User3 mentioned that the attention heat map presented various time granularities, for example daily, every 12 h, and so on.

## 4. Discussion

This paper proposed an interactive visualization system to assist in developing predictive models by integrating sequence mining, sequential data training, and visualization. According to our results, the system helps in defining a patient group that meets complex clinical criteria and provides high-performance and variable-level interpretation ability by using the long short-term memory (LSTM) model with an attention mechanism.

Firstly, similar to the transformer architecture, the attention mechanism contributed to the outstanding performance of the machine learning model [21,22], in addition to facilitating the interpretation of a substantial amount of medical data [16,23,24,25]. When the performance of a machine learning model is reasonable, it can be applied as a black box. However, model interpretation is essential when this is not the case [17,26]. If the reason for type-II errors generated by the model cannot be identified, it will be challenging to apply such a model in clinical practice. Although the current attention mechanism cannot explain all the situations entirely [27], we identified through experiments and interviews that assistive interpretation could be provided using our approach in clinical practice.

Owing to the complex nature of EHR data, visual and computational assistance is essential. Thus, we used data mining and visualization to facilitate the use of EHR data. We investigated whether the mined patterns can be used for conceptually representing clinical pathways, which entail the trends of disease progression, medical practice over time, and consequences of the medical practice. By interviewing experts, we confirmed that the pathways obtained in the use case could reflect complex clinical outcomes in clinical settings. Moreover, this study successfully labeled unplanned cardiac surgery data, which can be useful in hospital emergency departments. On the contrary, we found that most medical studies employ relatively simple outcomes that are identified based on code systems such as diagnostic codes or drug prescriptions [28,29]. Thus, this approach is expected to be particularly useful in developing an early diagnosis model for acute diseases having complex criteria, such as myocardial infarction [30].

In addition, most model development studies require the collaboration of experienced human experts. In particular, selecting features and defining training labels remains expensive and time consuming. Moreover, in this process, one should iteratively check that the predictive model is clinically relevant and reliable. Therefore, visualizing the attention of the model to aid in model interpretation facilitates the process of developing models with medical professionals and machine learning experts. User interviews have confirmed that this visualization method is informative, creative, and helps model developers to understand the inner workings of models.

The proposed system has two limitations: Firstly, the present study used timestamps recorded in the EHR data. However, improvement of the time resolution requires more accurate timestamps. If an event is recorded with only the date, we cannot compare such an event with one recorded with both the date and time. In addition, it was revealed that the timestamps of the EHR data may be inaccurate. These timestamps may represent the time when an event occurred, data were input, or medical staff ordered treatment. For events with no available date, we can use an approach where the timestamp is appended as a separate bucket after other data are extracted [31]. Secondly, the present study lacks out-of-sample testing. The full assessment of the generalization potential of the proposed system is possible only if this system is rigorously validated in varied patient groups and settings [16]. Although external validation can often be inadequate for this type of attempt owing to the low availability of datasets, the achievement of external validation before actual deployment is significant.

## 5. Conclusions

We applied the proposed system to develop the unplanned cardiac surgery prediction model. We demonstrated that event mining enables us to obtain clinically meaningful information from complex data. The proposed system, as a visualization assistant, enables the use of EHR data in various applications and facilitates the successful application of data-intensive techniques, such as deep learning and machine learning, to various patient groups. The system provides a novel and efficient way to evaluate clinical hypothesis, which can be developed as predictive models.

In future work, the increase in the amount of collected EHR data is expected to open new possibilities for gaining insight by using the temporal information of EHRs. These advances may promote the exploration of clinical hypotheses through the precise identification and verification of patient groups. The proposed architecture can be evaluated for other clinical use cases with the help of medical experts. This includes evaluating the qualitative aspects of pathway patterns based on the mining algorithm. The proposed attention model can also be reviewed with better clinical interpretation, and higher performance can be achieved. Furthermore, there are currently limitations to our approach because only a fixed database was applied; however, we plan to apply real-world data to construct a sustained continual learning [32] environment with regard to the data and the model.

## Figures and Tables

**Table 1:** Example sequence of a patient.

Patient ID, Subject_id	Hospital Admission ID, Hadm_id	Timestamp, ts	Event ID, Event_id	Event Value, Event_Value
18803	233	2189-12-24 7:15:00	2	1
18803	233	2189-12-24 7:15:00	6185	1
18803	254	2189-12-24 7:15:00	30	1
Sequence	(2), (6185), (30)			

**Table 2:** Results by the sequence mining parameter.

Minimum Support Rate (%)	Total Time (s)	Pattern Count
25	12.5	97
10	60.884	1094
5	154.066	8312
3	987.708	28,745

**Table 3:** Patterns strongly related to unplanned cardiac surgeries.

No.	Pattern	Support (%)
1	Chemistry: Troponin T ->CABG	0.051
2	Chemistry: Troponin T ->ECG ->CABG	0.046
3	Chemistry: Troponin T ->CABG ->Aspirin	0.046
4 *	Emergency ->Chemistry: Troponin T ->CABG	0.05
5	Emergency ->Ischemic_hd ->Chemistry: Troponin T ->CABG	0.049
6	Ischemic_hd ->Chemistry: Troponin T ->CABG	0.05
7	Emergency ->Chemistry: Troponin T ->ECG ->CABG	0.045
8	Emergency ->Chemistry: Troponin T ->CABG ->Aspirin	0.045
9	Emergency ->Ischemic_hd ->Chemistry: Troponin T ->ECG ->CABG	0.044
10	Emergency ->Ischemic_hd ->Chemistry: Troponin T ->CABG ->Aspirin	0.044
11	Ischemic_hd ->Chemistry: Troponin T ->ECG ->CABG	0.045
12	Ischemic_hd ->Chemistry: Troponin T ->CABG ->Aspirin	0.046

* A total of twelve patterns were found, among which the pattern of primary interest was pathway no. 4.
